# Hexa­aqua­zinc(II) dinitrate bis­[5-(pyridinium-3-yl)tetra­zol-1-ide]

**DOI:** 10.1107/S205698901801112X

**Published:** 2018-08-14

**Authors:** Ignacio Chi-Duran, Javier Enriquez, Andres Vega, Felipe Herrera, Dinesh Pratap Singh

**Affiliations:** a Department of Physics, University of Santiago, Av. Ecuador 3493, Estación Central, Santiago, Chile; b Departamento de Ciencias Quimicas, Universidad Nacional Andres Bello, Av Republica 275 3er Piso, Santiago, Region Metropolitana, Chile; c Millennium Institute for Research in Optics (MIRO), Chile

**Keywords:** crystal stryucture, pyridin-3-yl­tetra­zole, hexa­aqua­zinc(II) complex, π–π stacking, hydrogen-bonding

## Abstract

Hexa­aqua­zinc(II) dinitrate 5-(pyridinium-3-yl)tetra­zol-1-ide, [Zn(H_2_O)_6_](NO_3_)_2_·2C_6_H_5_N_5_, crystallizes in the space group *P* . The asymmetric unit contains one zwitterionic 5-(pyridinium-3-yl)tetra­zol-1-ide mol­ecule, one NO_3_^−^ anion and one half of a [Zn(H_2_O)_6_]^2+^ cation ( symmetry). The pyridinium and tetra­zolide rings in the zwitterion are nearly coplanar, with a dihedral angle of 5.4 (2)°. Several O—H⋯N and N—H⋯O hydrogen-bonding inter­actions exist between the [Zn(H_2_O)_6_]^2+^ cation and the N atoms of the tetra­zolide ring, and between the nitrate anions and the N—H groups of the pyridinium ring, respectively, giving rise to a three-dimensional network. The 5-(pyridinium-3-yl)tetra­zol-1-ide mol­ecules show parallel-displaced π–π stacking inter­actions; the centroid–centroid distance between adjacent tetra­zolide rings is 3.6298 (6) Å and that between the pyridinium and tetra­zolide rings is 3.6120 (5) Å.

## Abstract 2

In hexa­aqua­zinc(II) dinitrate 5-(pyridinium-3-yl)tetra­zol-1-ide, the pyridinium and tetra­zolide rings in the zwitterion are nearly coplanar. Several O—H⋯N and N—H⋯O hydrogen-bonding inter­actions exist between the [Zn(H_2_O)_6_]^2+^ cation and the N atoms of the tetra­zolide ring, and between the nitrate anions and the N—H groups of the pyridinium ring, respectively, giving rise to a three-dimensional network.

## Chemical context   

**Figure 1:**
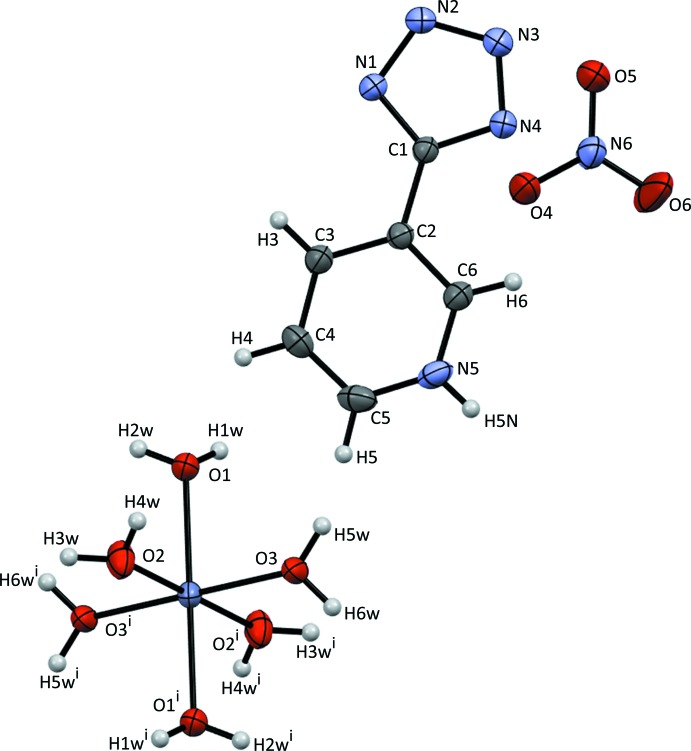
The mol­ecular structure of the asymmetric unit (plus the three water molecules of the hexaaquazinc cation generated by symmetry), showing the atom labelling and displacement ellipsoids drawn at the 50% probability level. [Symmetry code: (i) −*x*, 2 − *y*, 2 − *z*.]

Tetra­zole functional groups have attracted increased attention in recent years due to their use in drug design and their employment as isosteric subtitutents of carb­oxy­lic acids (Herr, 2002 ▸), as well as their ability to produce a large variety of metal–organic frameworks (MOFs) (Zhao *et al.*, 2008 ▸; Chi-Duran *et al.*, 2018 ▸). Push–pull tetra­zole complexes with both electron-donor and electron-acceptor substituents have shown efficient second-order nonlinear optical activity in powdered samples (Masahiko *et al.*, 1994 ▸), ferroelectric behaviour (Liu *et al.*, 2015 ▸) and strong photoluminescence (Zhang *et al.*, 2014 ▸). The *in-situ* synthesis of tetra­zole compounds can be realized by the Demko–Sharpless method, in which zinc salts catalyze the cyclo­addition reaction between sodium azide and nitrile compounds to form the tetra­zole ring (Demko & Sharpless, 2001 ▸). In this work, pyridyl­tetra­zole, synthesized at low pH using the Demko–Sharpless method, is cocrystallized in the presence of [Zn(H_2_O)_6_]^2+^ and NO_3_^−^ ions, to obtain the title compound (Fig. 1 ▸).
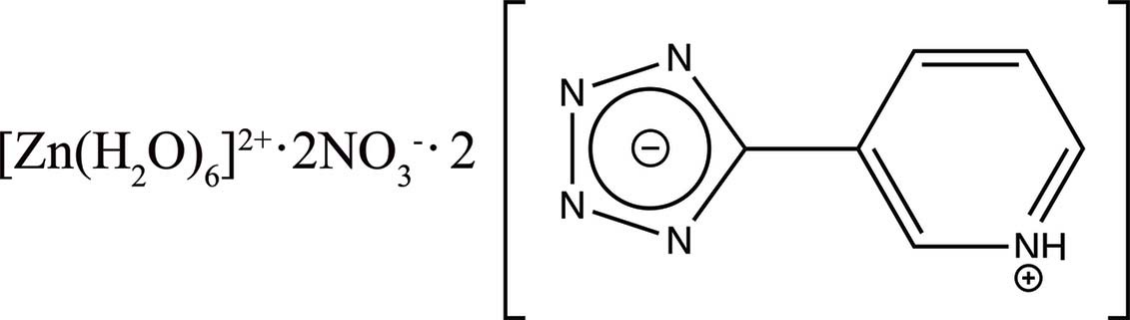


## Structural commentary   

**Figure 2:**
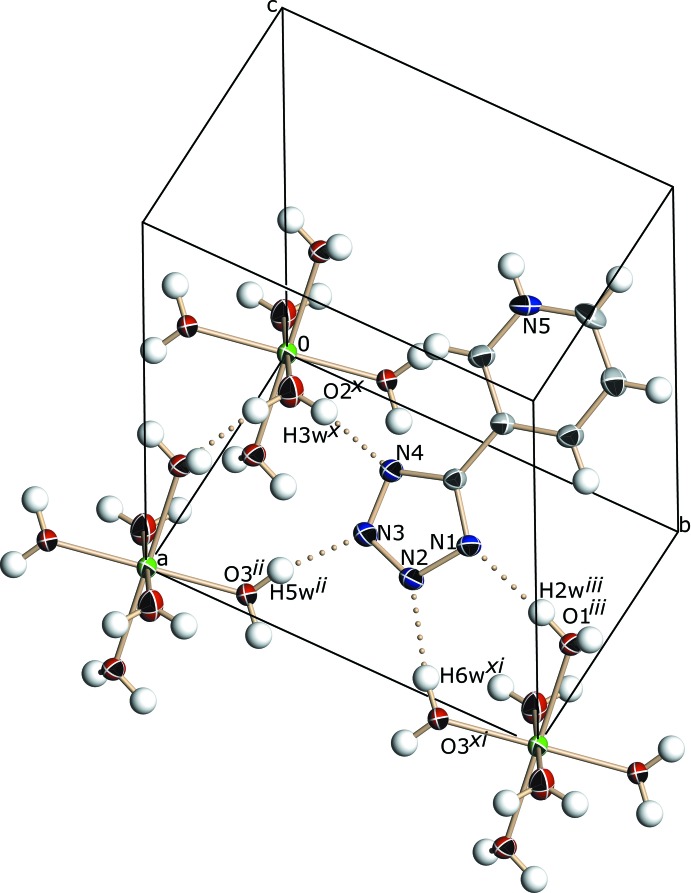
Partial crystal packing of the title compound, showing the hydrogen-bonding inter­actions between [Zn(H_2_O)_6_]^2+^ and the tetra­zolide ring. [Symmetry codes: (ii) −*x* + 1, −*y* + 1, −*z* + 1; (iii) −*x* + 1, −*y* + 2, −*z* + 1; (x) *x*, *y* − 1, *z* − 1; (xi) *x* + 1, *y*, *z* − 1.]

The asymmetric unit of the title compound is composed of one 5-(pyridinium-3-yl)tetra­zol-1-ide zwitterion, one NO_3_^−^ anion and one half of a [Zn(H_2_O)_6_]^2+^ cation. The hexa­aqua­zinc(II) complex exhibits regular octa­hedral geometry (Table 1 ▸), and the tetra­zolide and pyridinium rings of the zwitterion are close to being coplanar, with a dihedral angle of 5.4 (2)° (Fig. 2 ▸). The geometric parameters of the tetra­zolide ring are com­parable to those in other reported tetra­zole compounds (Mu *et al.*, 2010 ▸; Dai & Chen, 2011*a* ▸,*b* ▸). The H atom attached to the N atom of the pyridine ring could not be located in the Fourier density map. Therefore, the H atom was placed in accordance with similar reported structures containing [Mg(H_2_O)_6_]*X*_2_ (*X* = Cl^−^, Br^−^) cocrystallized with 5-(pyridinium-3-yl)tetra­zol-1-ide (Dai & Chen, 2011*a* ▸,*b* ▸).

## Supra­molecular features   

**Figure 3:**
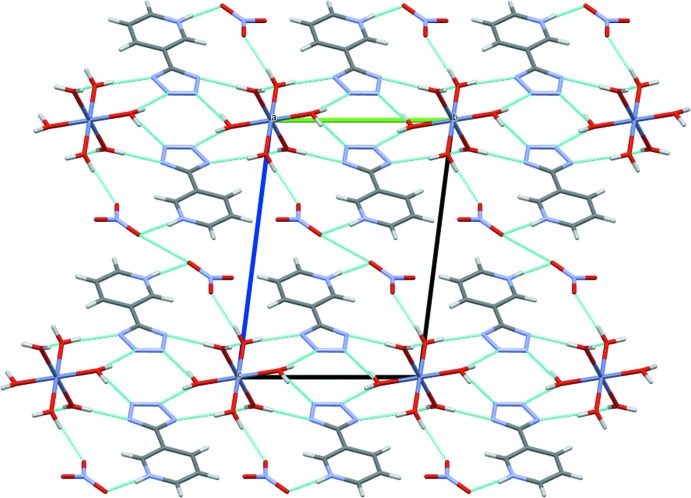
The crystal packing of the title compound, viewed along the [100] direction, showing O—H⋯N and N—H⋯O inter­actions (cyan lines).

**Figure 4:**
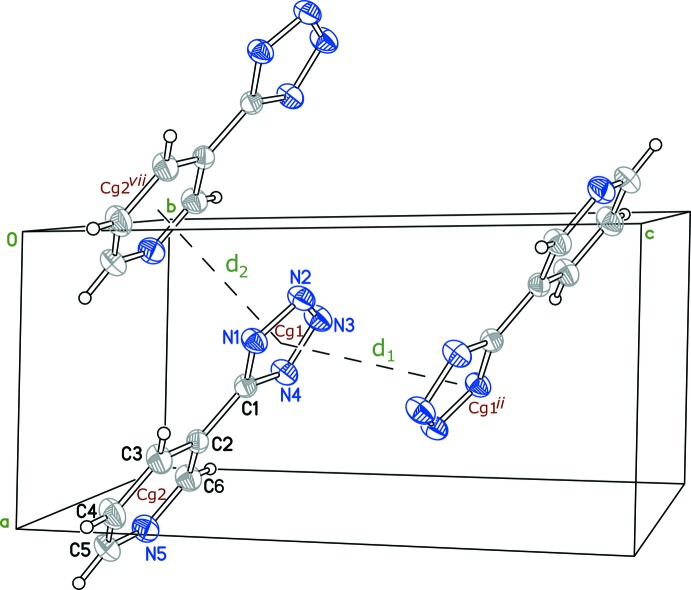
Partial crystal packing, showing π–π inter­actions between tetra­zole and pyridinium rings, with *d*_1_ = 3.6298 (6) Å and *d*_2_ = 3.6120 (5) Å. [Symmetry codes: (ii) −*x* + 1, −*y* + 1, −1 + *z*; (vii) *x* − 1, *y*, *z*.]

A three-dimensional network of hydrogen bonds involving the pyridinium–tetra­zolide zwitterions, hexa­aqua­zinc(II) com­plex cations and nitrate ions serves to hold the structure together (Table 2 ▸ and Fig. 3 ▸). The N atoms of the tetra­zole ring inter­act with the octa­hedral complex, [Zn(H_2_O)_6_]^2+^, through O—H⋯N hydrogen bonds, exhibiting *D*⋯*A* distances in the range 2.7446 (17)–2.8589 (17) Å. Additionally, the pyridinium ring is involved in N—H⋯O hydrogen bonding to nitrate atom O4, with an N⋯O distance of 2.7384 (18) Å. These inter­actions are shown in the crystal packing diagram (Fig. 3 ▸). The structure also shows parallel-displaced π–π stacking inter­actions, which arise from partial overlap between the tetra­zolide and pyridinium rings in adjacent zwitterions, and extend along the *a* axis parallel to the (010) plane. These parallel-displaced π–π inter­actions lead to inter­planar distances of 3.21 (1) and 3.10 (3) Å, and two centroid–centroid distances (Table 3 ▸). The centroid–centroid distance between the tetra­zolide groups is 3.6298 (6) Å and between the pyridinium and tetra­zolide rings is 3.6120 (5) Å (Table 3 ▸ and Fig. 4 ▸).

## Database survey   

We found two previously reported structures that are closely related to the title compound. They both involve a hexa­aqua­magnesium(II) cation with a halide counter-ion [chloride (Dai & Chen, 2011*b* ▸) or bromide (Dai & Chen, 2011*a* ▸)] cocrystallized in the presence of 5-(pyridinium-3-yl)tetra­zol-1-ide zwitterions (Dai & Chen, 2011*a* ▸,*b* ▸). There are more hydrogen-bonding inter­actions in our compound than in the [Mg(H_2_O)_6_]*X*_2_·2C_6_H_5_N_5_ structures, as more hexa­aqua­zinc(II) complexes can inter­act with the N atoms of the tetra­zole units. Parallel-displaced π–π stacking inter­actions occur in the title compound and in [Mg(H_2_O)_6_]*X*_2_·2C_6_H_5_N_5_. In [Mg(H_2_O)_6_]Cl_2_·2C_6_H_5_N_5_, the pyridinium–tetra­zolide zwitterions have alternating orientations in the supra­molecular arrangement, whereas in the title compound, the zwitterions are oriented in the same direction, allowing a possible coupling transition between dipole moments similar to J-aggregates (Spano, 2010 ▸).

## Synthesis and crystallization   

All the reactants and chemicals were purchased from Sigma Aldrich and utilized without further purification. A mixture of 3-cyano­pyridine (4 mmol), NaN_3_ (6 mmol) and ZnCl_2_ (2 mmol) were dissolved in 6 ml of distilled water. This mixture was transferred to a glass bottle and then heated at 378 K for 24 h. The pH was adjusted using a HNO_3_ (66%) solution immediately after mixing the reactants, and was monitored with a pH meter (pH2700 Oakton) until reaching a pH of 2.0. The reaction mixture was then cooled to 318 K and kept at this temperature for 16 h. The colourless block-shaped crystals obtained were washed with ethanol to give 353 mg (yield 30%) of the title compound.

## Refinement   

Crystal data, data collection and structure refinement details are summarized in Table 4 ▸. All H atoms bonded to C atoms were positioned geometrically and treated as riding atoms, using C—H = 0.93 Å and *U*_iso_(H) = 1.2*U*_eq_(C). Moreover, all H atoms in the hexa­aqua­zinc(II) complex were refined with a distance restraint of O—H = 0.85 Å and with *U*_iso_(H) = 1.5*U*_eq_(O).

## Supplementary Material

Crystal structure: contains datablock(s) I. DOI: 10.1107/S205698901801112X/cq2025sup1.cif

Structure factors: contains datablock(s) I. DOI: 10.1107/S205698901801112X/cq2025Isup3.hkl

CCDC reference: 1860162

Additional supporting information:  crystallographic information; 3D view; checkCIF report

## Figures and Tables

**Table 1:** Selected geometric parameters (Å, °)

Zn1—O3	2.0353 (11)	Zn1—O2^i^	2.1011 (12)
Zn1—O3^i^	2.0354 (11)	Zn1—O1^i^	2.1841 (11)
Zn1—O2	2.1011 (12)	Zn1—O1	2.1841 (11)
			
O3—Zn1—O2	90.01 (5)	O2—Zn1—O1^i^	92.10 (5)
O3—Zn1—O2^i^	89.99 (5)	O3—Zn1—O1	89.47 (4)
O3—Zn1—O1^i^	90.53 (4)	O2—Zn1—O1	87.90 (5)

Symmetry code: (i) 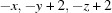.

**Table 2:** Hydrogen-bond geometry (Å, °)

*D*—H⋯*A*	*D*—H	H⋯*A*	*D*⋯*A*	*D*—H⋯*A*
O1—H1*W*⋯O5^ii^	0.85	1.96	2.8067 (17)	172
O1—H2*W*⋯N1^iii^	0.85	1.96	2.8029 (17)	173
O2—H3*W*⋯N4^iv^	0.85	2.02	2.8589 (17)	170
O2—H4*W*⋯O1^v^	0.85	2.08	2.9228 (17)	171
O3—H5*W*⋯N3^ii^	0.85	1.91	2.7446 (17)	168
O3—H6*W*⋯N1^vi^	0.85	2.72	3.4294 (17)	142
O3—H6*W*⋯N2^vi^	0.85	1.97	2.8076 (17)	169
N5—H5*N*⋯N6^vii^	0.82	2.61	3.344 (2)	149
N5—H5*N*⋯O4^vii^	0.82	1.92	2.7384 (18)	173
N5—H5*N*⋯O5^vii^	0.82	2.62	3.1347 (19)	123
C4—H4⋯O5^viii^	0.93	2.65	3.452 (2)	145
C5—H5⋯O4^ix^	0.93	2.52	3.292 (2)	141
C5—H5⋯O6^ix^	0.93	2.52	3.422 (3)	165
C6—H6⋯O5^vii^	0.93	2.41	3.047 (2)	126

Symmetry codes: (ii) 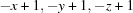; (iii) 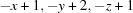; (iv) 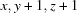; (v) 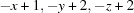; (vi) 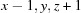; (vii) 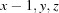; (viii) 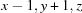; (ix) 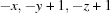.

**Table 3:** π–π stacking inter­action lengths (Å) *Cg*1 and *Cg*2 are the centroids of the C1/N1/N2/N3/N4 and C2–C6/N5 rings, respectively.

Centroid–centroid	Distance	Tetra­zolide inter­plane distance
*Cg*1–*Cg*1^ii^	3.6298 (6)	3.23 (1)
*Cg*1–*Cg*2^vii^	3.6120 (5)	3.10 (3)

Symmetry codes: (ii) −*x* + 1, −*y* + 1, −1 + *z*; (vii) *x* − 1, *y*, *z*.

**Table 4:** Experimental details

Crystal data	
Chemical formula	[Zn(H_2_O)_6_](NO_3_)_2_·2C_6_H_5_N_5_
*M*_r_	591.81
Crystal system, space group	Triclinic, *P* 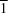
Temperature (K)	293
*a*, *b*, *c* (Å)	5.6582 (11), 8.4632 (16), 12.046 (2)
α, β, γ (°)	97.209 (2), 91.123 (2), 93.949 (2)
*V* (Å^3^)	570.67 (19)
*Z*	1
Radiation type	Mo *K*α
μ (mm^−1^)	1.16
Crystal size (mm)	0.49 × 0.21 × 0.09

Data collection	
Diffractometer	Bruker SMART CCD area detector
Absorption correction	Numerical (*SADABS*; Bruker, 2008 ▸)
*T*_min_, *T*_max_	0.742, 0.903
No. of measured, independent and observed [*I* > 2σ(*I*)] reflections	4429, 2217, 2132
*R*_int_	0.013
(sin θ/λ)_max_ (Å^−1^)	0.617

Refinement	
*R*[*F* ^2^ > 2σ(*F* ^2^)], *wR*(*F* ^2^), *S*	0.022, 0.054, 1.08
No. of reflections	2217
No. of parameters	198
No. of restraints	13
H-atom treatment	H atoms treated by a mixture of independent and constrained refinement
Δρ_max_, Δρ_min_ (e Å^−3^)	0.37, −0.35

Computer programs: *SMART* and *SAINT* (Bruker, 2008 ▸), *SHELXTL* (Sheldrick, 2008 ▸) and *SHELXL2014* (Sheldrick, 2015 ▸).

## supplementary crystallographic information

### Crystal data

**Table:**

[Zn(H_2_O)_6_](NO_3_)_2_·2C_6_H_5_N_5_	*Z* = 1
*M**_r_* = 591.81	*F*(000) = 304
Triclinic, *P*1	*D*_x_ = 1.722 Mg m^−^^3^
*a* = 5.6582 (11) Å	Mo *K*α radiation, λ = 0.71073 Å
*b* = 8.4632 (16) Å	Cell parameters from 7831 reflections
*c* = 12.046 (2) Å	θ = 2.8–29.5°
α = 97.209 (2)°	µ = 1.16 mm^−^^1^
β = 91.123 (2)°	*T* = 293 K
γ = 93.949 (2)°	Block, colorless
*V* = 570.67 (19) Å^3^	0.49 × 0.21 × 0.09 mm

### Data collection

**Table:**

Bruker SMART CCD area detector diffractometer	2132 reflections with *I* > 2σ(*I*)
Radiation source: sealed tube	*R*_int_ = 0.013
phi and ω scans	θ_max_ = 26.0°, θ_min_ = 2.4°
Absorption correction: numerical (SADABS; Bruker, 2008)	*h* = −6→6
*T*_min_ = 0.742, *T*_max_ = 0.903	*k* = −10→10
4429 measured reflections	*l* = −14→14
2217 independent reflections	

### Refinement

**Table:**

Refinement on *F*^2^	Hydrogen site location: mixed
Least-squares matrix: full	H atoms treated by a mixture of independent and constrained refinement
*R*[*F*^2^ > 2σ(*F*^2^)] = 0.022	*w* = 1/[σ^2^(*F*_o_^2^) + (0.0254*P*)^2^ + 0.1986*P*] where *P* = (*F*_o_^2^ + 2*F*_c_^2^)/3
*wR*(*F*^2^) = 0.054	(Δ/σ)_max_ < 0.001
*S* = 1.08	Δρ_max_ = 0.37 e Å^−^^3^
2217 reflections	Δρ_min_ = −0.35 e Å^−^^3^
198 parameters	Extinction correction: SHELXL2014 (Sheldrick, 2015), Fc^*^=kFc[1+0.001xFc^2^λ^3^/sin(2θ)]^-1/4^
13 restraints	Extinction coefficient: 0.009 (2)

### Special details

**Table:**

Geometry. All esds (except the esd in the dihedral angle between two l.s. planes) are estimated using the full covariance matrix. The cell esds are taken into account individually in the estimation of esds in distances, angles and torsion angles; correlations between esds in cell parameters are only used when they are defined by crystal symmetry. An approximate (isotropic) treatment of cell esds is used for estimating esds involving l.s. planes.

### Fractional atomic coordinates and isotropic or equivalent isotropic displacement parameters (Å^2^)

**Table:**

	*x*	*y*	*z*	*U*_iso_*/*U*_eq_	
Zn1	0.0000	1.0000	1.0000	0.02186 (9)	
O1	0.25606 (19)	1.00502 (12)	0.86692 (9)	0.0269 (2)	
H1W	0.226 (4)	0.9463 (19)	0.8050 (8)	0.044 (6)*	
H2W	0.303 (3)	1.0972 (10)	0.8515 (16)	0.041 (5)*	
O2	0.2699 (2)	1.10732 (13)	1.11255 (11)	0.0345 (3)	
H3W	0.307 (4)	1.2067 (5)	1.1282 (17)	0.048 (6)*	
H4W	0.401 (2)	1.065 (3)	1.116 (2)	0.065 (7)*	
O3	0.0981 (2)	0.78032 (12)	1.02590 (10)	0.0293 (2)	
H5W	0.187 (3)	0.725 (2)	0.9825 (14)	0.051 (6)*	
H6W	−0.008 (3)	0.7116 (19)	1.0423 (19)	0.059 (7)*	
N1	0.5987 (2)	0.68143 (14)	0.16630 (10)	0.0246 (3)	
N2	0.7365 (2)	0.58336 (15)	0.10516 (11)	0.0270 (3)	
N3	0.6508 (2)	0.43523 (15)	0.10431 (11)	0.0296 (3)	
N4	0.4544 (2)	0.43180 (14)	0.16488 (11)	0.0269 (3)	
N5	−0.0918 (2)	0.58146 (17)	0.38266 (11)	0.0315 (3)	
H5N	−0.180 (3)	0.5136 (17)	0.4067 (15)	0.042 (5)*	
C1	0.4273 (2)	0.58463 (16)	0.20195 (12)	0.0212 (3)	
C2	0.2376 (2)	0.64009 (17)	0.27458 (11)	0.0218 (3)	
C3	0.2065 (3)	0.80168 (18)	0.30257 (13)	0.0284 (3)	
H3A	0.3075	0.8778	0.2748	0.034*	
C4	0.0250 (3)	0.8491 (2)	0.37189 (14)	0.0334 (4)	
H4	0.0048	0.9570	0.3914	0.040*	
C5	−0.1243 (3)	0.7363 (2)	0.41142 (14)	0.0345 (4)	
H5	−0.2470	0.7668	0.4578	0.041*	
C6	0.0827 (3)	0.53073 (19)	0.31686 (13)	0.0279 (3)	
H6	0.0997	0.4219	0.2996	0.033*	
N6	0.6295 (3)	0.22071 (16)	0.38657 (12)	0.0353 (3)	
O4	0.5953 (2)	0.34960 (14)	0.44531 (11)	0.0442 (3)	
O5	0.8117 (2)	0.21012 (15)	0.33009 (11)	0.0431 (3)	
O6	0.4892 (4)	0.1062 (2)	0.38679 (19)	0.1001 (8)	

### Atomic displacement parameters (Å^2^)

**Table:**

	*U*^11^	*U*^22^	*U*^33^	*U*^12^	*U*^13^	*U*^23^
Zn1	0.02070 (14)	0.01556 (13)	0.02905 (15)	0.00093 (8)	0.00394 (9)	0.00145 (9)
O1	0.0295 (6)	0.0203 (5)	0.0304 (6)	−0.0002 (4)	0.0092 (4)	0.0015 (4)
O2	0.0292 (6)	0.0230 (6)	0.0485 (7)	−0.0009 (5)	−0.0073 (5)	−0.0031 (5)
O3	0.0283 (6)	0.0193 (5)	0.0419 (6)	0.0056 (4)	0.0149 (5)	0.0063 (5)
N1	0.0232 (6)	0.0217 (6)	0.0282 (6)	−0.0018 (5)	0.0064 (5)	0.0013 (5)
N2	0.0232 (6)	0.0257 (7)	0.0315 (7)	0.0011 (5)	0.0082 (5)	0.0013 (5)
N3	0.0282 (7)	0.0241 (6)	0.0360 (7)	0.0024 (5)	0.0095 (6)	0.0006 (5)
N4	0.0268 (7)	0.0199 (6)	0.0333 (7)	−0.0005 (5)	0.0086 (5)	0.0006 (5)
N5	0.0275 (7)	0.0374 (8)	0.0295 (7)	−0.0062 (6)	0.0086 (6)	0.0067 (6)
C1	0.0217 (7)	0.0197 (7)	0.0214 (7)	−0.0017 (5)	0.0015 (5)	0.0014 (5)
C2	0.0218 (7)	0.0228 (7)	0.0199 (7)	−0.0015 (5)	0.0018 (5)	0.0002 (5)
C3	0.0311 (8)	0.0239 (7)	0.0285 (8)	−0.0041 (6)	0.0076 (6)	−0.0004 (6)
C4	0.0373 (9)	0.0279 (8)	0.0332 (8)	0.0044 (7)	0.0079 (7)	−0.0049 (6)
C5	0.0283 (8)	0.0457 (10)	0.0284 (8)	0.0044 (7)	0.0092 (6)	−0.0015 (7)
C6	0.0279 (8)	0.0255 (8)	0.0297 (8)	−0.0030 (6)	0.0055 (6)	0.0033 (6)
N6	0.0398 (8)	0.0279 (7)	0.0346 (8)	−0.0094 (6)	0.0107 (6)	−0.0048 (6)
O4	0.0508 (8)	0.0301 (6)	0.0480 (7)	−0.0067 (5)	0.0237 (6)	−0.0080 (5)
O5	0.0428 (7)	0.0333 (6)	0.0502 (8)	−0.0024 (5)	0.0194 (6)	−0.0063 (6)
O6	0.0959 (14)	0.0607 (11)	0.1238 (16)	−0.0531 (10)	0.0672 (13)	−0.0446 (11)

### Geometric parameters (Å, º)

**Table:**

Zn1—O3	2.0353 (11)	N4—C1	1.3345 (19)
Zn1—O3^i^	2.0354 (11)	N5—C6	1.339 (2)
Zn1—O2	2.1011 (12)	N5—C5	1.339 (2)
Zn1—O2^i^	2.1011 (12)	N5—H5N	0.8201 (11)
Zn1—O1^i^	2.1841 (11)	C1—C2	1.463 (2)
Zn1—O1	2.1841 (11)	C2—C6	1.381 (2)
O1—H1W	0.8500	C2—C3	1.391 (2)
O1—H2W	0.8499	C3—C4	1.385 (2)
O2—H3W	0.8499	C3—H3A	0.9300
O2—H4W	0.8499	C4—C5	1.367 (2)
O3—H5W	0.8499	C4—H4	0.9300
O3—H6W	0.8501	C5—H5	0.9300
N1—C1	1.3384 (18)	C6—H6	0.9300
N1—N2	1.3405 (18)	N6—O6	1.210 (2)
N2—N3	1.3113 (18)	N6—O5	1.2485 (18)
N3—N4	1.3421 (18)	N6—O4	1.2525 (18)
			
O3—Zn1—O3^i^	180.0	N2—N3—N4	109.65 (12)
O3—Zn1—O2	90.01 (5)	C1—N4—N3	104.64 (12)
O3^i^—Zn1—O2	89.99 (5)	C6—N5—C5	122.90 (14)
O3—Zn1—O2^i^	89.99 (5)	C6—N5—H5N	117.6 (14)
O3^i^—Zn1—O2^i^	90.01 (5)	C5—N5—H5N	119.5 (14)
O2—Zn1—O2^i^	180.0	N4—C1—N1	111.53 (13)
O3—Zn1—O1^i^	90.53 (4)	N4—C1—C2	124.52 (13)
O3^i^—Zn1—O1^i^	89.47 (4)	N1—C1—C2	123.93 (13)
O2—Zn1—O1^i^	92.10 (5)	C6—C2—C3	118.26 (14)
O2^i^—Zn1—O1^i^	87.90 (5)	C6—C2—C1	119.94 (13)
O3—Zn1—O1	89.47 (4)	C3—C2—C1	121.80 (13)
O3^i^—Zn1—O1	90.53 (4)	C4—C3—C2	119.93 (14)
O2—Zn1—O1	87.90 (5)	C4—C3—H3A	120.0
O2^i^—Zn1—O1	92.10 (5)	C2—C3—H3A	120.0
O1^i^—Zn1—O1	180.0	C5—C4—C3	119.67 (15)
Zn1—O1—H1W	118.9 (14)	C5—C4—H4	120.2
Zn1—O1—H2W	115.7 (13)	C3—C4—H4	120.2
H1W—O1—H2W	107.0 (18)	N5—C5—C4	119.30 (15)
Zn1—O2—H3W	126.7 (15)	N5—C5—H5	120.3
Zn1—O2—H4W	120.1 (17)	C4—C5—H5	120.3
H3W—O2—H4W	104 (2)	N5—C6—C2	119.93 (15)
Zn1—O3—H5W	123.7 (14)	N5—C6—H6	120.0
Zn1—O3—H6W	118.5 (15)	C2—C6—H6	120.0
H5W—O3—H6W	103 (2)	O6—N6—O5	120.33 (15)
C1—N1—N2	104.70 (12)	O6—N6—O4	120.02 (15)
N3—N2—N1	109.48 (11)	O5—N6—O4	119.63 (13)
			
C1—N1—N2—N3	0.11 (16)	N1—C1—C2—C3	7.1 (2)
N1—N2—N3—N4	−0.01 (17)	C6—C2—C3—C4	0.5 (2)
N2—N3—N4—C1	−0.09 (17)	C1—C2—C3—C4	−179.80 (14)
N3—N4—C1—N1	0.17 (17)	C2—C3—C4—C5	−0.7 (3)
N3—N4—C1—C2	−178.53 (13)	C6—N5—C5—C4	0.5 (3)
N2—N1—C1—N4	−0.18 (16)	C3—C4—C5—N5	0.2 (3)
N2—N1—C1—C2	178.53 (13)	C5—N5—C6—C2	−0.7 (2)
N4—C1—C2—C6	5.4 (2)	C3—C2—C6—N5	0.2 (2)
N1—C1—C2—C6	−173.17 (14)	C1—C2—C6—N5	−179.53 (13)
N4—C1—C2—C3	−174.32 (15)		

Symmetry code: (i) −*x*, −*y*+2, −*z*+2.

### Hydrogen-bond geometry (Å, º)

**Table:**

*D*—H···*A*	*D*—H	H···*A*	*D*···*A*	*D*—H···*A*
O1—H1*W*···O5^ii^	0.85	1.96	2.8067 (17)	172
O1—H2*W*···N1^iii^	0.85	1.96	2.8029 (17)	173
O2—H3*W*···N4^iv^	0.85	2.02	2.8589 (17)	170
O2—H4*W*···O1^v^	0.85	2.08	2.9228 (17)	171
O3—H5*W*···N3^ii^	0.85	1.91	2.7446 (17)	168
O3—H6*W*···N1^vi^	0.85	2.72	3.4294 (17)	142
O3—H6*W*···N2^vi^	0.85	1.97	2.8076 (17)	169
N5—H5*N*···N6^vii^	0.82	2.61	3.344 (2)	149
N5—H5*N*···O4^vii^	0.82	1.92	2.7384 (18)	173
N5—H5*N*···O5^vii^	0.82	2.62	3.1347 (19)	123
C4—H4···O5^viii^	0.93	2.65	3.452 (2)	145
C5—H5···O4^ix^	0.93	2.52	3.292 (2)	141
C5—H5···O6^ix^	0.93	2.52	3.422 (3)	165
C6—H6···O5^vii^	0.93	2.41	3.047 (2)	126

Symmetry codes: (ii) −*x*+1, −*y*+1, −*z*+1; (iii) −*x*+1, −*y*+2, −*z*+1; (iv) *x*, *y*+1, *z*+1; (v) −*x*+1, −*y*+2, −*z*+2; (vi) *x*−1, *y*, *z*+1; (vii) *x*−1, *y*, *z*; (viii) *x*−1, *y*+1, *z*; (ix) −*x*, −*y*+1, −*z*+1.
